# Insulin alleviates degradation of skeletal muscle protein by inhibiting the ubiquitin-proteasome system in septic rats

**DOI:** 10.1186/1476-9255-8-13

**Published:** 2011-06-03

**Authors:** Qiyi Chen, Ning Li, Weiming Zhu, Weiqin Li, Shaoqiu Tang, Wenkui Yu, Tao Gao, Juanjuan Zhang, Jieshou Li

**Affiliations:** 1 Department of General Surgery, Jinling Hospital, Medical College of Nanjing University, Nanjing 210002, Jiangsu Province, China

## Abstract

Hypercatabolism is common under septic conditions. Skeletal muscle is the main target organ for hypercatabolism, and this phenomenon is a vital factor in the deterioration of recovery in septic patients. In skeletal muscle, activation of the ubiquitin-proteasome system plays an important role in hypercatabolism under septic status. Insulin is a vital anticatabolic hormone and previous evidence suggests that insulin administration inhibits various steps in the ubiquitin-proteasome system. However, whether insulin can alleviate the degradation of skeletal muscle protein by inhibiting the ubiquitin-proteasome system under septic condition is unclear. This paper confirmed that mRNA and protein levels of the ubiquitin-proteasome system were upregulated and molecular markers of skeletal muscle proteolysis (tyrosine and 3-methylhistidine) simultaneously increased in the skeletal muscle of septic rats. Septic rats were infused with insulin at a constant rate of 2.4 mU.kg^-1^.min^-1 ^for 8 hours. Concentrations of mRNA and proteins of the ubiquitin-proteasome system and molecular markers of skeletal muscle proteolysis were mildly affected. When the insulin infusion dose increased to 4.8 mU.kg^-1^.min^-1^, mRNA for ubiquitin, E2-14 KDa, and the C2 subunit were all sharply downregulated. At the same time, the levels of ubiquitinated proteins, E2-14KDa, and the C2 subunit protein were significantly reduced. Tyrosine and 3-methylhistidine decreased significantly. We concluded that the ubiquitin-proteasome system is important skeletal muscle hypercatabolism in septic rats. Infusion of insulin can reverse the detrimental metabolism of skeletal muscle by inhibiting the ubiquitin-proteasome system, and the effect is proportional to the insulin infusion dose.

## Introduction

Muscle catabolism, resulting in muscle wasting and fatigue, is a characteristic metabolic response to sepsis[1-3]. Sepsis-induced muscle catabolism is mainly caused by increased protein breakdown, in particular myofibrillar protein breakdown, although reduced protein synthesis and inhibited amino acid transport contribute to the metabolic response. Muscle breakdown may impair the recovery in septic patients and increase the risk for pulmonary and thrombo-embolic complications when respiratory muscles and ambulation are affected[3-6]

Previous studies provided evidence that sepsis-induced muscle proteolysis is caused by increased protein breakdown, through the ubiquitin (Ub)-proteasome pathway[4-6]. In this pathway, Ub, which contains 76 amino acids, is conjugated to proteins destined for degradation by Ub-activating enzyme (E1), Ub-conjugating enzyme (E2), and Ub-ligase (E3) [1,7]. The 14-kDa ubiquitin-conjugating enzyme E2 has been proposed to be a regulation site for the Ub-proteasome proteolytic pathway in skeletal muscle[8]. This process is repeated as multiple Ub molecules are added to form a Ub chain. Ub-protein conjugates are recognized by a 26S proteasome complex, composed of two subproteasome complexes, a 19S regulatory particle, and a 20S catalytic particle. Ubiquitinated proteins are rapidly degraded by the proteasome in an ATP-dependent manner[2,9].

Insulin is an anabolic and anticatabolic hormone. When administered to healthy volunteers, it stimulates muscle protein synthesis and inhibits protein metabolism at the whole body level[10]. In addition, several studies have repeatedly demonstrated the beneficial effects of insulin treatment on protein wasting caused by different pathological conditions. For example, in burn-injured patients and in animal experiments, nitrogen balance is partially restored after continuous infusion of insulin[11]. In diabetic patients and diabetic rats, administration of insulin decreases or completely prevents the release of urinary 3-methylhistidine (3MH) [10]. In patients in intensive care unit, insulin administration reduces morbidity by preventing organ failure, as evidenced by a reduction in duration of mechanical ventilation[12]. However, the molecular mechanism by which insulin suppresses protein degradation remains poorly understood.

Previous evidence suggests that insulin deficiency results in activity of the Ub-proteasome system[13-15]. Insulin resistance causes muscle wasting by mechanisms that involve activation of the Ub-proteasome proteolytic pathway, causing muscle protein degradation[15,16]. Insulin administration can inhibit various steps of the Ub-proteasome system; for example, a hyperinsulinaemic euglycaemic clamp significantly reduced mRNA expression for theubiquitin system in rat skeletal muscle[17]. Fouzia Sadiq et al. showed that, in C2C12 myotubes, insulin administration was associated with downregulated expression of the Ub-proteasome pathway[18]. However, no reports on the influence of insulin on theUb-proteasome system under sepsis currently exist. In this study, we hypothesize that infusion of insulin would alleviate degradation of skeletal muscle protein by inhibiting the Ub-proteasome system in septic rats.

## Materials and methods

### Animals

This study used 44 adult male Sprague-Dawley rats, weighing 200 ± 20 g, from the animal center of Jinling Hospital. The Institutional Animal Care Committee approved the study protocol. The Association accredits the animal care facility for Assessment and Accreditation of Laboratory Animal Care. Rats were housed in mesh cages in a 25°C room, illuminated in 12:12-h light-dark cycles and acclimatized to their environment for 7 d before the study. They were provided with standard rodent chow and water ad libitum.

### Animal preparation

Rats were anesthetized intraperitoneally with phenobarbital sodium (60 mg/kg), and catheters (PE-50, PE-10; Becton-Dickinson, Sparks, MD) were implanted into the right jugular vein and the left carotid artery, as described previously[19]. The right jugular vein was used to infuse insulin and dextrose solution by micropump (proved by the Research Center for Analytical Instrument, Zhejiang University) and the left carotid artery was used to monitor blood glucose with an Elite glucometer (Bayer, Elkhart, IN). The catheters were filled with saline containing heparin sodium.

### Group distribution and insulin infusion strategy

After 5-6 days recovery, rats were fasted for 12 h and divided randomly into four groups as follows: control group (n = 12) LPS group (n = 12), low-dose insulin group (n = 12) and high-dose insulin group (n = 12). The sepsis model was mimicked by intraperitoneal injection with 10 mg/kg LPS (*Escherichia coli *serotype 055:B5, Sigma, St. Louis, MO). The low- or high-dose insulin group rats received a continuous infusion of insulin (Humulin R, Eli Lilly & Co., Indianapolis, IN) at a constant rate of 2.4 (low) or 4.8 (high) mU^-1^min^-1^kg^-1 ^for 8 hours after LPS stimulation. Blood glucose was maintained between 4.4-6.1 mmol/L by varying the infusion rate of a 50% dextrose solution. The LPS group was injected intraperitoneally with 10 mg/kg LPS only. The control group received an intraperitoneal injection with an equal volume of sterile saline only. Experiment were performed while the rats were awake and unrestrained.

At the end of the infusion, rats were killed with phenobarbital sodium. The extensor digitorum longus (EDL) was immediately excised to measure the proteolytic rate, and the gastrocnemius muscle was harvested and frozen in -80°C.

### Rate of protein turnover

To measure protein breakdown rates, freshly EDL muscle was fixed via the tendons to aluminum wire supports at resting length, and preincubated in oxygenated medium (95% O_2_-5% CO_2_): Krebs-Henseleit bicarbonate buffer (pH 7.4) containing 5 Mm glucose, 0.1 U/ml insulin, 0.17 mM leucine, 0.1 mM isoleucine, and 0.20 mM valine. After a 1 h preincubation, muscles were transferred to fresh medium of identical composition and incubated for a further 2 h with 0.5 mM cycloheximide. The degradation rates of total and myofibrillar proteins were determined by release in the medium of free tyrosine and 3-MH, respectively, and expressed as nanomoles of tyrosine/methylhistidine in medium per 2·h^-1^g·muscle^-1^. Muscle was also homogenized in 0.4 mM perchloric acid to determine tissue free tyrosine and 3-MH. The net production of free tyrosine was calculated as the amount of tyrosine released into the medium plus the increase in tissue free tyrosine during incubation. Net 3-MH production was calculated as the amount of 3-MH in the medium minus the decrease in tissue free 3-MH before and after incubation. Levels of both tyrosine and 3-MH in medium or tissue samples were determined by high-performance liquid chromatography (HPLC).

### RT-PCR RNA preparation and analysis of expression of ubiquitin-proteasome system genes

Total mRNA was extracted from gastrocnemius with TRIzol reagents (Life Technologies), and the mRNA concentration determined by ultraviolet light absorbency at 260 nm. Measurement of mRNA of the Ub-proteasome system components ubiquitin, 14-kDa Ub-conjugating enzyme (14-kDa E2), and proteasome subunit C2 was performed by semi-quantitative reverse transcriptase-polymerase chain reaction (RT-PCR). Before reverse transcription, 1-5 μg of total RNA was reverse transcribed at 42°C for 1 h using standard reagents of 50 μl of 1×RT, reverse transcriptase (RTase), and poly(dT)12-18 primer. RT mixtures were heated at 100°C for 10 min to inactivate RTase. PCR reactions were 50 μl of 1×PCR buffer with 5 μl of RT template, 200 nM each sense and antisense primers, 1 unit of *Taq *polymerase, and 200 μM each dNTPs. The reaction was covered with 30 μl of mineral oil, and PCR was performed in a DNA Thermal Cycler 480 (Perkin-Elmer, Norwalk, CT). After 94°Cfor 5 min, cycles were 94°C for 30 s, annealing at a product-specific temperature for 1 min, and 72°C for 1 min. The last cycle was followed by 5 min at 72°C. The number of amplification cycles was optimized for primer pairs to produce a densitometric result that correlated closely with the template. To determine the relative quantities of mRNA, 10 μl each of Ub, 14-kDa, proteasome subunit C2, and β-actin PCR products amplified from the same RT template were combined and electrophoresed on 2% agarose gel in Tris-acetate-EDTA buffer for 30 min. After ethidium bromide staining for 15 min, bands were measured for densitometry using Quantity One Analysis software. Relative Ub mRNA levels in the original RNA extracts fromthe skeletal muscle preparations were obtained by normalizaion to β-actin expression. Primer sequences were: Ubiquitin(200 bp): forward, 5'-TCTTCGTGAAGACCCTGACC-3'; reverse,5'-CAGGTGCAGGGTTGACTCTT-3'; E2-14KDa(221): forward, 5'-GTGCACCATCTGAAAACAA-3';reverse, 5'-ATCGGTTCTGCAGGATGTCT-3'; C2(256):forward, 5'-GGCTGCTCATTGCTGGTTAG-3'; reverse,5'-CCAACAATCCCAATGGAAAC-3'; β-actin(180): forward, 5'-TCCTGTGGCATCCACGAAACT-3', reverse, 5'-GGAGCAATGATCTTGATCTTC-3'.

### Western blot analysis

To detect proteins with conjugated Ub, E2-14KDa, and C2, myofibrillar and sarcoplasmic muscle proteins were prepared as described previously[2]. Myofibrillar and sarcoplasmic muscle protein were separated on SDS-polyacrylamide gels and transferred to nitrocellulose membranes. Membranes were blocked for 1 h in 5% (vol/vol) nonfat dry milk in TTNS (25 mM Tris-HCl [pH 7.5], 0.1% [vol/vol] Tween 20, 0.9% [wt/vol] NaCl). To detect conjugated Ub, myofibrillar proteins were incubated for 1 h with a 1:1000 diluted rabbit polyclonal anti-Ub antibody (DakoCytomation, Glostrup, Denmark). To quantify E2-14KDa and C2, sarcoplasmic proteins were incubated for 1 h with a 1:1000 diluted rabbit polyclonal anti-14-kDa E2 antibody and anti-C2 antibody (DakoCytomation, Glostrup, Denmark). After washing at room temperature, membranes were hybridized with the appropriate peroxidase-conjugated anti-IgG. Blots were washed four times with TTNS for 20 min, incubated in enhanced chemiluminescence reagent (Amersham Life Sciences), and exposed on radiographic film (Eastman-Kodak, Rochester, NY). Proteins were quantified by densitometry as above. Statistical analyses were carried out on data normalized to by β-actin.

### Statistical analyses

Data are expressed as means ± standard error (SE), and statistical analysis performed using ANOVA. All data were analyzed with SPSS software (Statistical Package for the Social Sciences, version 16.0, for Windows, SPSS, Chicago, IL). P *<*0.05 was considered significant.

## Results

### Proteolytic rate in extensor digital longus muscle

**Figure 1:**
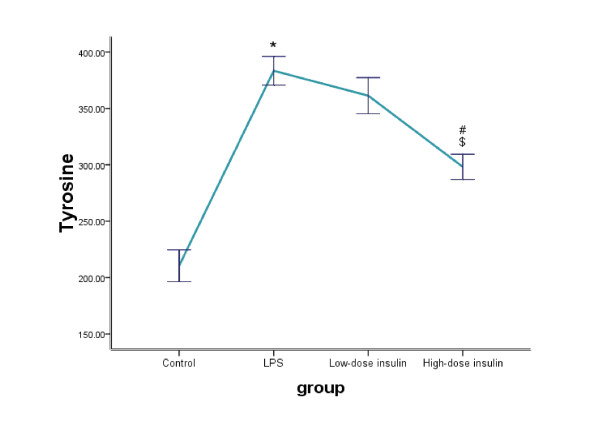
**Level of tyrosine in the medium or tissue samples determined by HPLC**. Data aremeans ± SE, and expressed as nanomoles of tyrosine in medium per 2·h-1 g·muscle-1. Comparison to control group, *P < 0.01; to LPS group, #P < 0.01; to low-dose insulin group, $P < 0.01.

**Figure 2:**
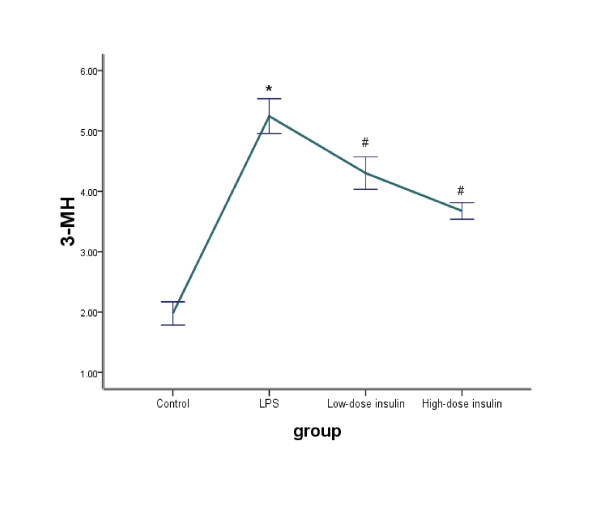
**Level of 3-MH in medium or tissue samples determined by HPLC**. Data are means ± SE, and expressed as nanomoles methylhistidine in medium per 2·h-1 g·muscle-1. Comparison to control group, *P < 0.01; to LPS group, #P < 0.01.

The proteolytic rate of skeletal muscle was measured as net release of tyrosine for total protein and 3-MH for myofibrillar protein. Compared to the control group, the rate of total protein proteolysis was significantly increased in the LPS group (210.49 ±14.09 vs. 383.4 ± 12.72, P < 0.01). Although release of tyrosine was affected slightly in the low-dose insulin group in sepsis rats (383.4 ± 12.72 vs. 361.3 ± 16.05, P = 0.26), when the infusion rate of insulin was increased to 4.8 mU.kg^-1^.min^-1^, the net release of tyrosine was significantly decreased compared to the LPS and low-dose insulin groups (298.21 ± 11.18 vs. 383.4 ± 12.72, P < 0.01; 298.21 ± 11.18 vs. 361.3 ± 16.05, P < 0.01). Similarly, compared to the control group, myofibrillar protein breakdown in the LPS treatment group was significantly increased (1.98 ± 0.19 vs. 5.25 ± 0.29, P < 0.01). With administration of 2.4 mU.kg^-1^.min^-1 ^insulin, the net release of 3-MH was decreased in sepsis rats (5.25 ± 0.29 vs. 4.3 ± 0.27, P <0.01). When the infusion rate of insulin was increased (4.8 mU.kg^-1^.min^-1^), the inhibition effect on the net release of 3-MH increased slightly compared to the low-dose insulin group (4.3 ± 0.27 vs. 3.67 ± 0.14, P = 0.069) (Figure 1, 2).

### Ubiquitin, E2-14KDa, and proteasome subunit C2 expression in gastrocnemius muscle

**Figure 3:**
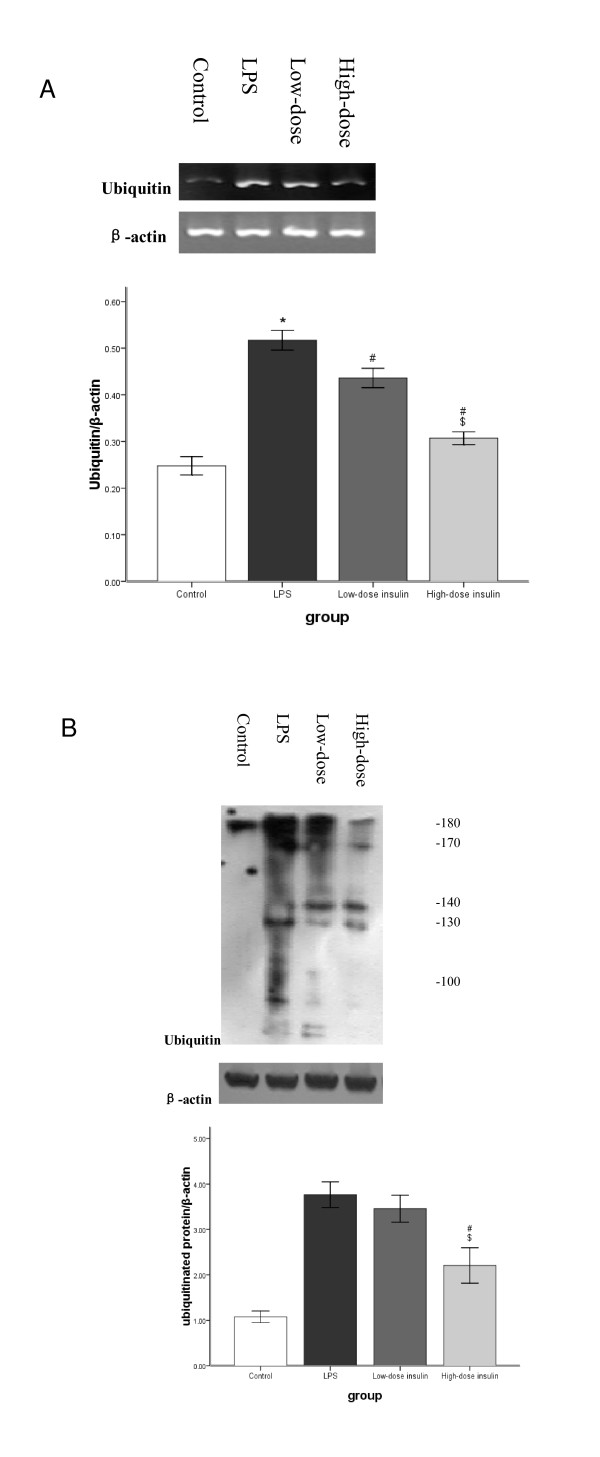
**A. mRNA for Ub in gastrocnemius muscles measured by semiquantitative RT-PCR normalized to β-actin**. Data are means ± SE. Compared to control group, *P < 0.05; to LPS group, #P < 0.05; to low-dose insulin group, $P < 0.05. **B**. Ubiquitinated protein analysed by western blot with protein levels quantified by densitometry and normalized to β-actin. Compared to control group, *P < 0.05; to LPS group, #P < 0.05; to low-dose insulin group, $P < 0.05.

**Figure 4:**
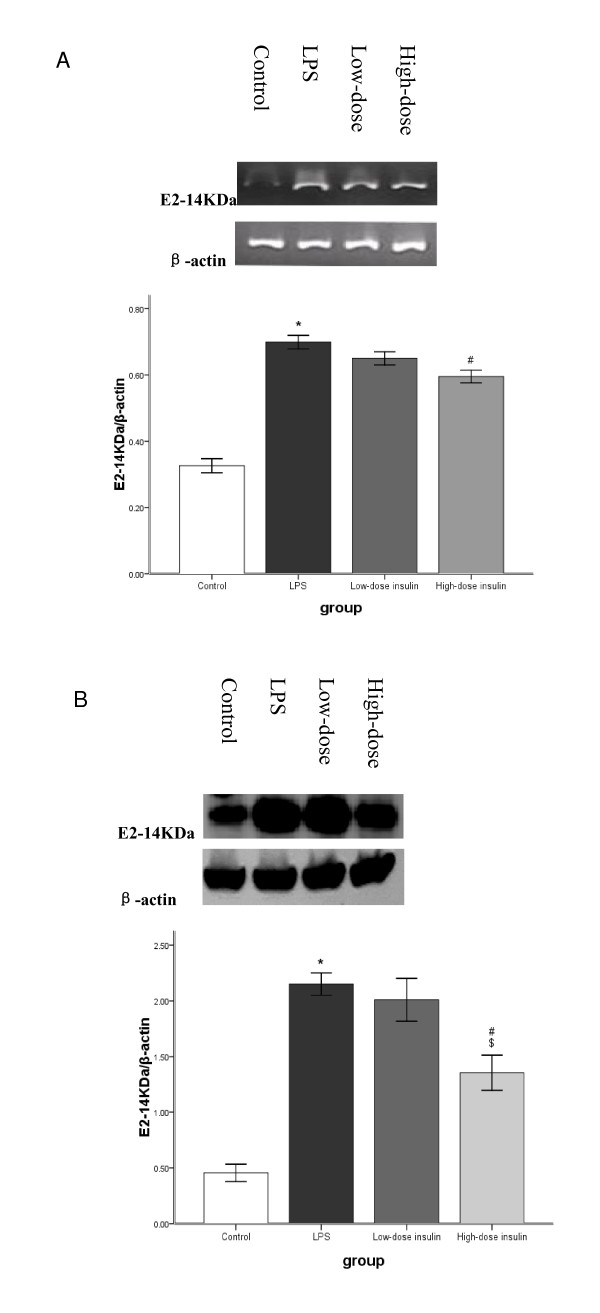
**A. mRNA for E2-14KDa in gastrocnemius muscle measured by semiquantitative RT-PCR with normalization to β-actin**. Data are means ± SE. Compared to control group, *P < 0.05; to LPS group, #P < 0.05. **B**. E2-14KDa protein was by western blot with protein levels quantified by densitometry and normalized to β-actin. Compared to control group, *P < 0.05; to LPS group, #P < 0.05; to low-dose insulin group, $P < 0.05.

**Figure 5:**
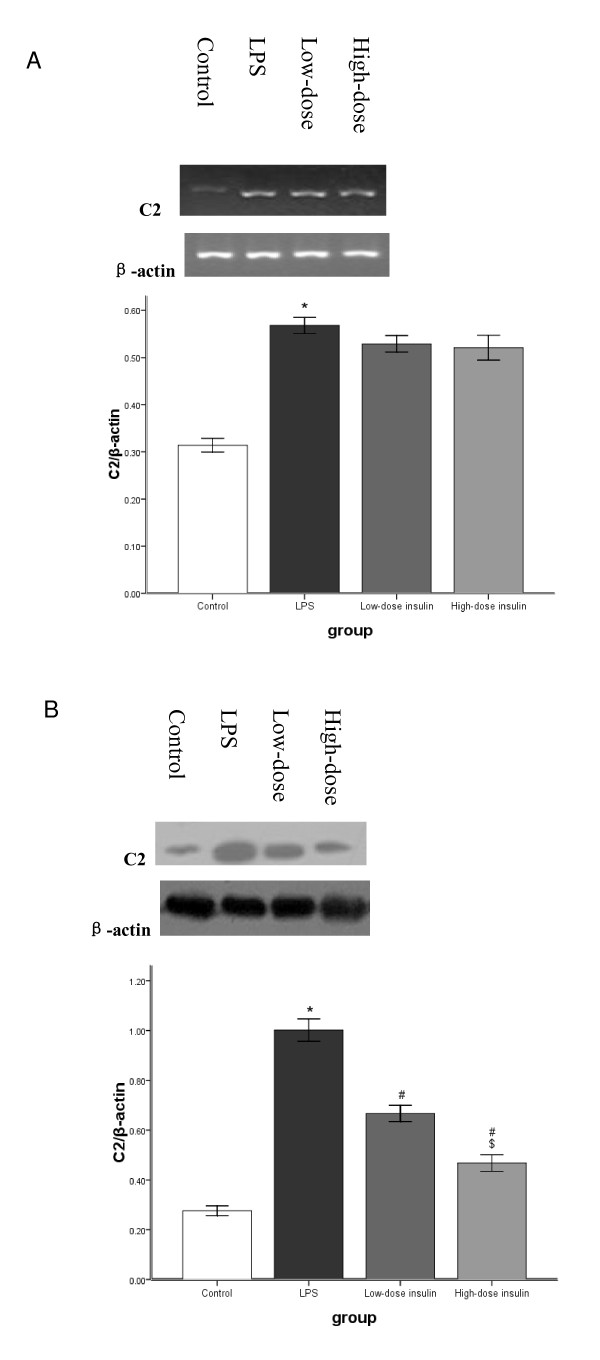
**A. mRNA for proteasome subunit C2 in gastrocnemius muscle measured by semiquantitative RT-PCR with normalization to β-actin**. Data are means ± SE. Compared to control group, *P < 0.05. **B**. Proteasome subunit C2 protein by western blot with protein levels quantified by densitometry and normalized to β-actin. Compared to control group, *P < 0.05; to LPS group, #P < 0.05; to low-dose insulin group, $P < 0.05.

RT-PCR analysis indicated that Ub, E2-14KDa, and C2 mRNA in gastrocnemius muscle was upregulated significantly after LPS injection. However, after infusion of insulin, Ub mRNA levels in septic rats decreased gradually and was dependent on the insulin infusion dos (Figure 3A). Although low-dose insulin had no notable influence on E2-14KDa mRNA expression, when the insulin infusion rate was increased to 4.8 mU.kg^-1^.min^-1^, E2-14KDa mRNA expression was downregulated (Figure 4A). However, insulin infusion had no influence on C2 mRNA expression in septic rats (Figure 5A).

### Western blot analysis

Western blot analysis showed that LPS pretreatment increased Ub conjugation, E2-14KDa, and the proteasome subunit C2 proteins in gastrocnemius muscle, and Ub conjugation was mainly to high-molecular weight proteins. Although low-dose insulin infusion had no influence on Ub conjugation, when the insulin infusion was increased to 4.8 mU.kg^-1^.min^-1^, levels of proteins conjugated to Ub in septic rats decreased compared to the LPS and low-dose insulin groups (Figure 3B). Compared to the LPS group, no difference was observed after low-dose insulin infusion; however, when the insulin infusion was 4.8 mU.kg^-1^.min^-1^, the concentration of E2-14KDa protein decreased compared to the LPS and low-dose insulin groups (Figure 4B). After insulin infusion, the levels of the proteasome subunit C2 in septic rats decreased gradually (Figure 5B).

## Discussion

Severe injury, infection, and other critical illnesses are associated with excessive loss of body protein. Muscle catabolism, resulting in muscle wasting and fatigue, is a characteristic metabolic response to sepsis[1-3]. Sepsis-induced muscle catabolism is mainly caused by increased protein breakdown, in particular myofibrillar protein breakdown, although reduced protein synthesis and inhibited amino acid transport contribute to the metabolic response. Muscle breakdown may impair recovery in septic patients and increase the risk for ulmonary and thrombo-embolic complications when respiratory muscles and ambulation are affected[3-6]. Ub Ub A loss of greater than 10% body protein contributes significantly to morbidity and debility[20]. Methods to reduce the catabolic response in skeletal muscle during sepsis, therefore, have great clinical significance.

Many studies have confirmed the importance of the Ub-proteasome system for breaking down intracellular proteins during pathophysiologic conditions, for example, severe sepsis, burn, diabetes, or trauma[2,21-25]. In this study, we used a classical rat LPS model of intraperitoneal injection with 10 mg/kg LPS to mimic sepsis. After induction of sepsis for 8 h, we found that expression of the genes for Ub, E2-14KDa, and the 20S proteasome subunit C2 were upregulated significantly. At the same time, the concentration of ubiquitinated proteins, E2-14KDa, and C2, increased notably in the LPS group compared to the control group. Molecular markers of skeletal muscle proteolysis (tyrosine and 3-MH) increased prominently.

Our results are consistent with other studies. For example, Chai, et al.[25] suggested that after intraperitoneal injection of 10 mg/kg LPS, mRNA for Ub, E2-14KDa, and C2 were upregulated significantly compared to normal control rats, while the rate of total protein breakdown and myofibrillar proteolysis increased. Van Beneden et al. [26] similarly found that Ub and E2-14KDa mRNA increased in rat tibialis anterior muscles after LPS injection. Hobler et al.[27] suggested that the Ub-proteasome system E214kDa increased 70% in EDL in septic rats induced by cecal ligation and puncture. Subsequently, they discovered a three- to four-fold increase in mRNA levels for Ub and the 20s proteasome in muscle tissue from septic patients, concomitant with increased muscle levels of phenylalanine and 3-MH[8]. These data support our results that the Ub-proteasome system was activated in skeletal muscle under septic conditions.

The function of the Ub-proteasome system is independent of the amount of protein consumed. Consequently, simple nutritional supplementation would not be expected to attenuate muscle catabolism[28]. Thus, developing new therapeutic approaches for treating muscle wasting is important, especially for hypermetabolism patients. Currently, several studies suggest that insulin can inhibit the activation of the Ub-proteasome system. For example, a low level of plasma insulin triggers protein degradation in muscle through activation of the Ub-proteasome pathway[13], while higher levels downregulate the expression of the 14-kDa E2 conjugating enzyme *in vitro *[14]. *In vivo*, a 6-hour hyperinsulinaemic euglycaemic clamp significantly reduced Ub mRNA in fast twitch and mixed skeletal muscle. Observations in hepatoma cells show that insulin regulates anticatabolic activity by decreasing Ub mediated proteasomal activity[17]. However, under sepsis conditions, whether insulin also inhibits the expression of the Ub is not currently known.

In our study, we hypothesized than continuous insulin infusion would alleviate degradation of skeletal muscle protein by inhibiting the Ub-proteasome system under sepsis conditions. After intraperitoneal injection with LPS, low-and high-dose insulin group animals received a continuous infusion of insulin at 2.4 mU.kg^-1^.min^-1 ^or 4.8 mU.kg^-1^.min^-1^, and blood glucose was controlled to 4.4-6.1 mmol/L. After 8 h, we found that mRNA for Ub, and the protein concentration of the proteasome subunit C2 in the low-dose insulin group were significantly higher than in the LPS group. At the same time, 3-MH was also reduced, but the concentration of tyrosine and other mRNA and protein levels of the Ub system changed only slightly. When the infusion dose of insulin was increased to 4.8 mU.kg^-1^.min^-1^, the Ub mRNA level was further reduced compared to the low-dose insulin group. Compared to the LPS group, E2-14KDa mRNA was downregulated prominently. Although insulin infusion had no influence on C2 mRNA expression, C2 protein levels were significantly decreased and the extent of C2 protein decrease was proportional to the insulin dose. The concentration of ubiquitinated proteins was also downregulated. Because high-dose insulin infusion reduced the activity of the Ub system, the release of tyrosine and 3-MH in EDL also decreased. These findings, to some extent, suggest that infusion of insulin alleviates degradation of skeletal muscle protein by inhibiting the Ub-proteasome system, and the effect is proportional to the insulin infusion dose.

Several possible mechanisms may result in insulin regulation of Ub-proteasome activity. Several animal experiments and clinical evidence suggest that in diabetes, the PI3K-Akt pathway plays a key role in inhibiting the activity of the Ub system[29-31]. However, whether PI3K-Akt has the same effect under sepsis is not yet known. Our preliminary experiments showed that after administering LY294002, an inhibitor of the PI3K-Akt pathway, the inhibiting effect of insulin was clearly decreased. Insulin resistance is common in septic conditions, resulting in a relative lack of insulin *in vivo*[29,32,33]. Hu et al. [14] found that insulin deficiency activated the Ub-proteasome system, resulting in cardiac muscle protein catabolism in diabetes mellitus. They also found that insulin resistance accelerates muscle protein degradation by activation of the Ub-proteasome[34]. Other studies suggested administration of insulin significantly reduced Ub mRNA [14-16]. However in our study, the relationship between insulin resistance and activation of Ub system were not confirmed. Our preliminary results show that insulin significantly inhibited the release of inflammatory cytokines such as TNF-α, IL-1 and IL-6 in septic patients [35]. These inflammatory cytokines are key factors for activity of the Ub-proteasome system [36]. Thus, insulin may inhibit the activity of the Ub system by inhibiting inflammatory cytokines. However, the correlation between insulin, cytokines and the Ub system remains to be further investigated. Another possibility is that insulin may inhibit the proteasome through an associated protein, IDE. The catalytic properties of the proteasome can vary widely, depending on its association with regulatory proteins [37]. Previous studies showed that insulin inhibits the proteasome *in vitro *and in cultured cells. Removal of IDE from the extracts or introduction of a neutralizing antibody into cells results in a loss of insulin regulation of the proteasome [38,39].

In conclusion, our results suggested that insulin administration to septic rats can inhibit the Ub-proteasome system with an effect proportional to the insulin infusion dose. These findings may provide a new therapeutic strategy for hypercatabolism patients under septic conditions or other critical illnesses.

## Competing interests

The authors declare that they have no competing interests.

## Authors' contributions

QC and TG participated in collection of data. NL and WY conceived and designed this study. WL and JZ did the statistical analysis. QC and WY wrote the first draft of the paper and JL commented on the draft. All other authors provided comments and approved the final manuscript.

## References

[B1] Hasselgren PO Role of the ubiquitin-proteasome pathway in sepsis-induced muscle catabolism Mol Biol Rep 1999 26 1-2 71 6 10363650 10.1023/a:1006916206260

[B2] Lin SY Chen WY Lee FY Huang CJ Sheu WH Activation of ubiquitin-proteasome pathway is involved in skeletal muscle wasting in a rat model with biliary cirrhosis: potential role of TNF-alpha Am J Physiol Endocrinol Metab 2005 288 3 E493 501 15522995 10.1152/ajpendo.00186.2004

[B3] Tiao G Hobler S Wang JJ Sepsis is associated with increased mRNAs of the ubiquitin-proteasome proteolytic pathway in human skeletal muscle J Clin Invest 1997 99 2 163 8 10.1172/JCI119143 9005983 PMC507782

[B4] Hasselgren PO Fischer JE The ubiquitin-proteasome pathway: review of a novel intracellular mechanism of muscle protein breakdown during sepsis and other catabolic conditions Ann Surg 1997 225 3 307 16 10.1097/00000658-199703000-00011 9060588 PMC1190682

[B5] Klaude M Fredriksson K Tjader I Proteasome proteolytic activity in skeletal muscle is increased in patients with sepsis Clin Sci (Lond) 2007 112 9 499 506 10.1042/CS20060265 17117920

[B6] Minnaard R Wagenmakers AJ Combaret L Ubiquitin-proteasome-dependent proteolytic activity remains elevated after zymosan-induced sepsis in rats while muscle mass recovers Int J Biochem Cell Biol 2005 37 10 2217 25 10.1016/j.biocel.2005.05.002 15955721

[B7] Dardevet D Sornet C Taillandier D Savary I Attaix D Grizard J Sensitivity and protein turnover response to glucocorticoids are different in skeletal muscle from adult and old rats. Lack of regulation of the ubiquitin-proteasome proteolytic pathway in aging J Clin Invest 1995 96 5 2113 9 10.1172/JCI118264 7593595 PMC185859

[B8] Hobler SC Wang JJ Williams AB Sepsis is associated with increased ubiquitinconjugating enzyme E214k mRNA in skeletal muscle Am J Physiol 1999 276 2 Pt 2 R468 73 9950926 10.1152/ajpregu.1999.276.2.R468

[B9] Rajan VR Mitch WE Muscle wasting in chronic kidney disease: the role of the ubiquitin proteasome system and its clinical impact Pediatr Nephrol 2008 23 4 527 35 10.1007/s00467-007-0594-z 17987322 PMC2259254

[B10] Solomon V Madihally S Yarmush M Toner M Insulin suppresses the increased activities of lysosomal cathepsins and ubiquitin conjugation system in burn-injured rats J Surg Res 2000 93 1 120 6 10.1006/jsre.2000.5958 10945952

[B11] Solomon V Madihally S Mitchell RN Yarmush M Toner M Antiproteolytic action of insulin in burn-injured rats J Surg Res 2002 105 2 234 42 10.1006/jsre.2002.6355 12121712

[B12] Van den Berghe G Wilmer A Hermans G Intensive insulin therapy in the medical ICU N Engl J Med 2006 354 5 449 61 10.1056/NEJMoa052521 16452557

[B13] Price SR Bailey JL Wang X Muscle wasting in insulinopenic rats results from activation of the ATP-dependent, ubiquitin-proteasome proteolytic pathway by a mechanism including gene transcription J Clin Invest 1996 98 8 1703 8 10.1172/JCI118968 8878419 PMC507607

[B14] Mitch WE Bailey JL Wang X Jurkovitz C Newby D Price SR Evaluation of signals activating ubiquitin-proteasome proteolysis in a model of muscle wasting Am J Physiol 1999 276 5 Pt 1 C1132 8 10329962 10.1152/ajpcell.1999.276.5.C1132

[B15] Hu J Klein JD Du J Wang XH Cardiac muscle protein catabolism in diabetes mellitus: activation of the ubiquitin-proteasome system by insulin deficiency Endocrinology 2008 149 11 5384 90 10.1210/en.2008-0132 18653708 PMC2734490

[B16] Wang X Hu Z Hu J Du J Mitch WE Insulin resistance accelerates muscle protein degradation: Activation of the ubiquitin-proteasome pathway by defects in muscle cell signaling Endocrinology 2006 147 9 4160 8 10.1210/en.2006-0251 16777975

[B17] Wing SS Banville D 14-kDa ubiquitin-conjugating enzyme: structure of the rat gene and regulation upon fasting and by insulin Am J Physiol 1994 267 1 Pt 1 E39 48 8048511 10.1152/ajpendo.1994.267.1.E39

[B18] Sadiq F Hazlerigg DG Lomax MA Amino acids and insulin act additively to regulate components of the ubiquitin-proteasome pathway in C2C12 myotubes BMC Mol Biol 2007 8 23 10.1186/1471-2199-8-23 17371596 PMC1845170

[B19] Sugita H Kaneki M Tokunaga E Inducible nitric oxide synthase plays a role in LPS-induced hyperglycemia and insulin resistance Am J Physiol Endocrinol Metab 2002 282 2 E386 94 11788371 10.1152/ajpendo.00087.2001

[B20] Ling PR Lydon E Qu Z Frederich RC Bistrian BR Metabolic effects of insulin and insulin-like growth factor-I in endotoxemic rats during total parenteral nutrition feeding Metabolism 2000 49 5 611 5 10.1016/S0026-0495(00)80036-0 10831171

[B21] Tisdale MJ The ubiquitin-proteasome pathway as a therapeutic target for muscle wasting J Support Oncol 2005 3 3 209 17 15915823

[B22] Wing SS Control of ubiquitination in skeletal muscle wasting Int J Biochem Cell Biol 2005 37 10 2075 87 10.1016/j.biocel.2004.11.011 16125111

[B23] Wray CJ Mammen JM Hershko DD Hasselgren PO Sepsis upregulates the gene expression of multiple ubiquitin ligases in skeletal muscle Int J Biochem Cell Biol 2003 35 5 698 705 10.1016/S1357-2725(02)00341-2 12672461

[B24] Breen HB Espat NJ The ubiquitin-proteasome proteolysis pathway: potential target for disease intervention JPEN J Parenter Enteral Nutr 2004 28 4 272 7 10.1177/0148607104028004272 15291411

[B25] Chai J Wu Y Sheng ZZ Role of ubiquitin-proteasome pathway in skeletal muscle wasting in rats with endotoxemia Crit Care Med 2003 31 6 1802 7 10.1097/01.CCM.0000069728.49939.E4 12794423

[B26] Olberding KE Kelley ML Butler RA Van Beneden RJ A HECT E3 ubiquitin-protein ligase with sequence similarity to E6AP does not target p53 for degradation in the softshell clam (*Mya arenaria*) Mutat Res 2004 552 1-2 61 71 15288542 10.1016/j.mrfmmm.2004.06.004

[B27] Tiao G Hobler S Wang JJ Sepsis is associated with increased mRNAs of the ubiquitin-proteasome proteolytic pathway in human skeletal muscle J Clin Invest 1997 99 2 163 8 10.1172/JCI119143 9005983 PMC507782

[B28] Tisdale MJ The ubiquitin-proteasome pathway as a therapeutic target for muscle wasting J Support Oncol 2005 3 3 209 17 15915823

[B29] Glass DJ PI3 kinase regulation of skeletal muscle hypertrophy and atrophy Curr Top Microbiol Immunol 2010 346 267 78 10.1007/82_2010_78 20593312

[B30] Stitt TN Drujan D Clarke BA The IGF-1/PI3K/Akt pathway prevents expression of muscle atrophy-induced ubiquitin ligases by inhibiting FOXO transcription factors Mol Cell 2004 14 3 395 403 10.1016/S1097-2765(04)00211-4 15125842

[B31] Lee SW Dai G Hu Z Regulation of muscle protein degradation: coordinated control of apoptotic and ubiquitin-proteasome systems by phosphatidylinositol 3 kinase J Am Soc Nephrol 2004 15 6 1537 45 10.1097/01.ASN.0000127211.86206.E1 15153564

[B32] Griesdale DE de Souza RJ van DRM Intensive insulin therapy and mortality among critically ill patients: a meta-analysis including NICE-SUGAR study data CMAJ 2009 180 8 821 7 19318387 10.1503/cmaj.090206 PMC2665940

[B33] Yu WK Li WQ Li N Li JS Influence of acute hyperglycemia in human sepsis on inflammatory cytokine and counterregulatory hormone concentrations World J Gastroenterol 2003 9 8 1824 7 12918129 10.3748/wjg.v9.i8.1824 PMC4611552

[B34] Wang X Hu Z Hu J Du J Mitch WE Insulin resistance accelerates muscle protein degradation: Activation of the ubiquitin-proteasome pathway by defects in muscle cell signaling Endocrinology 2006 147 9 4160 8 10.1210/en.2006-0251 16777975

[B35] Yu WK Li WQ Wang XD Influence and mechanism of a tight control of blood glucose by intensive insulin therapy on human sepsis Zhonghua Wai Ke Za Zhi 2005 43 1 29 32 15774170

[B36] Llovera M Garcia-Martinez C Agell N Lopez-Soriano FJ Argiles JM TNF can directly induce the expression of ubiquitin-dependent proteolytic system in rat soleus muscles Biochem Biophys Res Commun 1997 230 2 238 41 10.1006/bbrc.1996.5827 9016756

[B37] DeMartino GN Slaughter CA Regulatory proteins of the proteasome Enzyme Protein 1993 47 4-6 314 24 7697129 10.1159/000468689

[B38] Duckworth WC Bennett RG Hamel FG A direct inhibitory effect of insulin on a cytosolic proteolytic complex containing insulin-degrading enzyme and multicatalytic proteinase J Biol Chem 1994 269 40 24575 80 7929129

[B39] Hamel FG Bennett RG Harmon KS Duckworth WC Insulin inhibition of proteasome activity in intact cells Biochem Biophys Res Commun 1997 234 3 671 4 10.1006/bbrc.1997.6693 9175773

